# Application of a Heat- and Steam-Generating Sheet Increases Peripheral Blood Flow and Induces Parasympathetic Predominance

**DOI:** 10.1155/2011/965095

**Published:** 2011-04-26

**Authors:** Yoshinao Nagashima, Michihito Igaki, Atsushi Suzuki, Shuichi Tsuchiya, Yoshimi Yamazaki, Michiko Hishinuma, Sachiko Oh-ishi, Masataka Majima

**Affiliations:** ^1^Tokyo Research Laboratories, Kao Corporation, 2-1-3, Bunka, Sumida-ku, Tokyo 131-8501, Japan; ^2^Department of Pharmacology, Kitasato University School of Medicine, 1-15-1, Kitasato Sagamihara, Kanagawa 228-8555, Japan; ^3^St. Luke's College of Nursing, 10-1, Akashi-cho, Chuo-ku, Tokyo 104-0044, Japan; ^4^Kitasato University, 5-9-1, Shirokane, Minato-ku, Tokyo 108-8642, Japan

## Abstract

To promote the practical application of a Japanese traditional medical treatment, such as hot compresses, we developed a plaster-type warming device consisting of a heat- and steam-generating sheet (HSG sheet). First, we tested its effects when applied to the anterior abdominal wall or lumbar region of women complaining of a tendency towards constipation. Application of the sheet to either region produced a feeling of comfort in the abdomen, as assessed by a survey of the subjects. The significant increases in the total hemoglobin observed in these regions suggested an increase in peripheral blood flow, and significant increases in the HF component on ECG and in the amplitude of gastric motility suggested parasympathetic predominance. We concluded that application of the HSG sheet improves the peripheral hemodynamics and autonomic regulation, induces a feeling of comfort in the abdomen, and provides a beneficial environment for the improvement of gastrointestinal movements.

## 1. Introduction

The application of hot or warm compresses to the abdomen or lumbar region is often used in Japan as a traditional medicine to produce improvements in the physical condition of patients. For example, during the aftercare of patients who have undergone an abdominal operation, such nursing care stimulates blood flow and releases flatus and/or feces. However, it is difficult to maintain constant warmth and to control the amount of steam released onto the pack application area with the use of hot towels.

Therefore, we developed a heat- and steam-generating sheet (HSG sheet), a new heating material composed of a sheet of paper embedded with immobilized iron powder and containing an excess of water wrapped in unwoven cloth [1]. When applied, this sheet generates heat and steam and maintains the temperature between the sheet and the skin at about 38.5°C for approximately 5 hours. Hosono et al. applied an HSG sheet for 7 consecutive days (5 hours or more per day) to the hypogastric region of 16 healthy female students suffering from constipation (mean age: 20 years) and reported that HSG sheet treatment promoted the normalization of intestinal function and alleviated constipation [2]. 

However, the precise mechanism by which the application of the HSG sheet produces an improvement in intestinal function is not yet known, and the physiological effects of applying this sheet remain to be elucidated. Therefore, the present study was undertaken to evaluate the effects of the application of an HSG sheet to the anterior abdominal or lumbar region on peripheral hemodynamics, autonomic nervous activity, and gastrointestinal motility in volunteers. During preliminary tests of this sheet involving its application to a limited number of healthy women, we obtained promising effects. Therefore, we decided to test its effects precisely in healthy volunteer women that demonstrated a tendency towards constipation.

## 2. Materials and Methods

### 2.1. Heat- and Steam-Generating Sheet (HSG Sheet)

The HSG sheet tested was composed of two rectangular pads (each 12 × 10 cm) joined horizontally to each other. From the outermost to the innermost layer that is placed against the anterior abdominal region or lumbar region, this sheet is composed of a nonpermeable layer, a layer of heat-generating components (iron, active carbon, physiological saline, etc.), a permeable layer, and an unwoven cloth layer (a polyporous sheet composed of three-dimensionally overlapped textile). Oxidation of iron powder in the presence of oxygen in the ambient air and water generates 402 kJ of heat per mol of iron, and this heat causes the water contained in the heat-generating layer to steam. This heat causes steam to be produced from the water contained in the heat-generating layer. The skin of the anterior abdominal region or lumbar region is exposed to the steam passing through the permeable layer and the unwoven cloth. The temperature between the sheet and the skin is maintained at about 38.5°C (posing no risk of burns; the sheet is a medical device falling under the category of hot packs for home use), and heat and steam (amount of steam: about 200 mg/10 min) are generated from this sheet.

### 2.2. Subjects

The study involved 10 women aged 20 to 39 years (mean age: 30.3 ± 6.0 years) who satisfied the following requirements: being almost healthy but demonstrating a tendency towards or being concerned about constipation; a defecation score of 5 or higher (full score: 16) during the previous month (i.e., modest constipation) according to the Japanese version of the constipation assessment scale (CAS) developed by McMillan and Williams [3] (which was translated into Japanese after partial modifications), which corresponds to a constipation severity that would attract attention during nursing care, only being able to perform bowel movements once in 4 days, no requirement for laxatives; a BMI (body mass index; body weight (kg)/height (m)^2^) of less than 25, and being in the low body temperature phase (within 2 weeks of the start of menstruation) of a regular menstrual cycle. Each subject was asked to remain seated in a chair reclined at an angle of 60 degrees for 20 minutes in a room maintained at a temperature of 23.0°C and a relative humidity of 45.0% for the purpose of acclimation. Then, either an HSG sheet fitted with a belt (HSG sheet belt) or a sham belt was applied to the anterior abdominal region (the center of the anterior abdominal region around the umbilicus) or the lumbar region (the area bounded above by the iliac crests and below by the lowest ribs). In Japan, warming of the lumbar region with hot towels (lumbar hot pack) was clinically introduced based on a report in the 1970s that gas and/or feces were released from the stoma when back care with steam-warmed towels (application of steam-warmed towels to the back) was performed as part of nursing care to promote blood circulation and clean the skin in patients after open abdominal surgery (the reference is not shown, because it is written in Japanese). These findings suggested that lumbar warming with a hot towel (hot pack) in patients with abdominal distension or constipation alleviates symptoms such as the sensation of abdominal fullness by promoting intestinal peristalsis, and this technique began to be used extensively. Therefore, we applied an HSG sheet to this lumbar region. The anterior abdominal region to which HSG sheet was applied was located at the same spinal cord segment level as that of lumbar region. The study was designed as a random-order study involving 3 test conditions: (1) control: a sham belt was applied to the anterior abdominal region or lumbar region, (2) HSG-Ab: an HSG sheet was applied to the anterior abdominal region, and (3) HSG-Lum: an HSG sheet was applied to the lumbar region. The study was carried out with the approval of the Ethics Committee of St Luke's College of Nursing. All individuals participating in this study were informed about the design and method of the study and were only enrolled after they had provided written consent. All the procedures in this clinical study were carried out in accordance with the ethical principles set forth in the Declaration of Helsinki.

### 2.3. Protocol for Measurement

**Figure 1:**
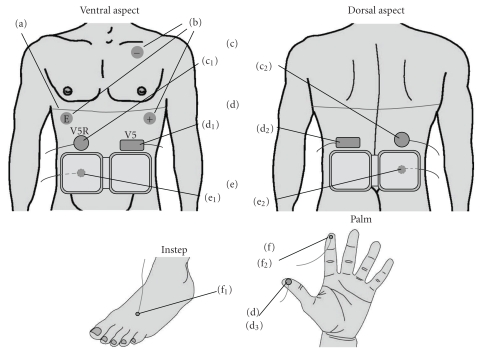
Sites of placement of the sensors. Upper left: ventral aspect of the upper half of the body, upper right: dorsal aspect of the upper half of the body, lower left: instep, lower right: palm, (a) a band for respiration, (b) leads for ECG (electrocardiogram), (c) sensors for subcutaneous tissue temperature; (c_1_) anterior abdominal region, (c_2_) lumbar region, (d) sensors for the tissue total hemoglobin level and tissue blood oxygen saturation; (d_1_) anterior abdominal muscle issue, (d_2_) lumbar muscle tissue, (d_3_) fingertip skin, (e) sensors for skin surface temperature beneath the HSG sheet; (e_1_) anterior abdominal region, (e_2_) lumbar region, (f) sensors for skin surface temperature; (f_1_) instep, (f_2_) fingertips.

**Figure 2:**
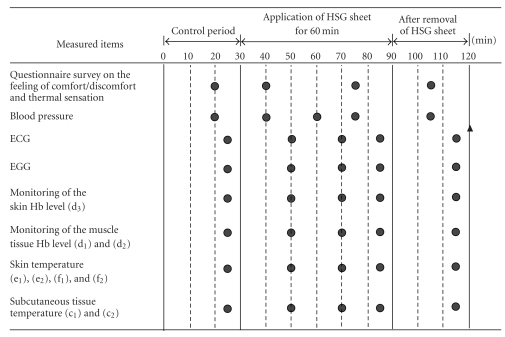
Experimental protocol. Random-order study involving 3 conditions (application of the HSG sheet to the anterior abdominal region, application of the HSG sheet to the lumbar region, and application of a sham belt to the anterior abdominal region and/or lumbar region). The following were measured or assessed: blood pressure, ECG (electrocardiography), EGG (electrogastrography), skin tissue total hemoglobin level and skin tissue oxygen saturation, muscle tissue total hemoglobin level and muscle tissue oxygen saturation, skin tissue temperature beneath the HSG sheet, skin temperature, subcutaneous tissue temperature, feeling of comfort/discomfort in the abdominal, and perception of thermal sensation. The time schedule was as follows. During the first 30 minutes, each subject remained still (the control period). Then, the HSG sheet belt or the sham belt was applied for 60 minutes (the period marked with shading). After removal of the belt, the subjects again remained still for 30 minutes. Points of measurement are indicated by ●. ▴: measured at 119 minutes.

The following measurements were conducted until the end of the study period, after the respective devices used for the measurements were attached to the subjects, as shown in Figure 1. An electrocardiographic (b) record was obtained with the V5 chest lead (Polymate AP1124, Digitex Lab. Co., Ltd., Tokyo, Japan); respiration (a) was monitored by the respiration band method (TR-751T R610, Nihon Kohden Corporation, Tokyo, Japan); percutaneous electrogastrography (Figure 4) was performed (EG, Nipro Corporation, Osaka, Japan); the tissue hemoglobin level at the finger tip (d_3_) was monitored by near-infrared spectroscopy, based on the modified Lambert-Beer law (PAS-500 PSD-22, Biomedical Science Inc., Kanazawa, Japan); the muscle tissue hemoglobin level in the anterior abdominal region (d_1_) and lumbar region (d_2_) was monitored by near-infrared spectroscopy [4–7], based on the modified Lambert-Beer law (PAS-500 PSD-30, Biomedical Science Inc., Kanazawa, Japan); skin temperature (e_1_, e_2_, f_1_, f_2_) was measured with a contact-type thermocouple temperature sensor (LT-6, Gram Corporation, Saitama, Japan); subcutaneous tissue temperature (c_1_, c_2_) was measured by the heat flow compensation method [8, 9] (Coretemp CM-210, Terumo Corporation, Tokyo, Japan). The measurements were carried out according to the time schedule shown in Figure 2. During the first 30 minutes, each subject remained still (control period). Then, the HSG sheet belt or the sham belt was applied for 60 minutes. After removal of the respective belts, the subjects were again requested to remain seated and still for 30 minutes. Blood pressure was measured by the oscillometric method [10] (DINAMAP PRO 100, GE Healthcare, Little Chalfont, UK) at 20 minutes after the start of the test, at 10, 30, and 45 minutes during and after application of the HSG sheet or sham belt, and at 15 and 29 minutes after the removal of the belts. Feeling and thermal sensation (perception of skin temperature) by the individual subjects was examined by the self-reporting method (five-category scale) at 20 minutes after the start of the test, at 10 and 45 minutes during and after application of the HSG sheet or sham belt, and at 15 minutes after the removal of the belts.

### 2.4. Answers on the Feeling of Comfort/Discomfort in the Abdomen and Perception of Thermal Sensation

Each subject was asked to answer a question on the level of comfort/discomfort in the abdomen and the fingertip thermal sensation on a five-category scale, and the responses were scored and totaled for analysis. The five-category scoring system for the feeling of comfort/discomfort in the abdomen was: comfortable (5), slightly comfortable (4), neutral (3), slightly uncomfortable (2), and uncomfortable (1). The five-category scoring system for fingertip thermal sensation was warm (5), slightly warm (4), neutral (3), slightly cold (2), and cold (1). The questionnaire survey on the feeling of comfort/discomfort in the abdomen and fingertip thermal sensation was conducted at 4 points of time, that is, 20 minutes after acclimation, 10 and 45 minutes during and after application of the HSG sheet belt or sham belt, and 15 minutes after the removal of either belt. The mean score for 10 subjects was analyzed.

### 2.5. Tissue Total Hemoglobin Level and Oxygen Saturation

**Figure 3:**
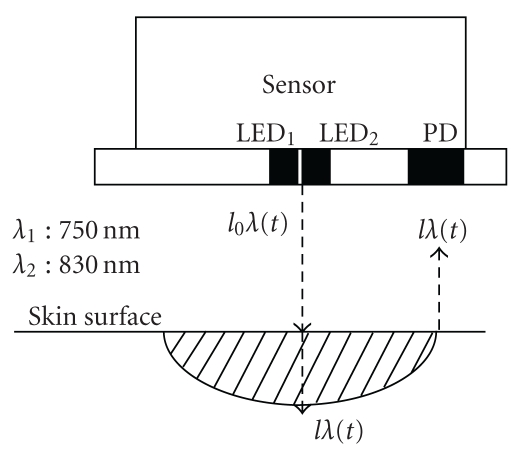
Cross-section of the sensor for measurement of the tissue total hemoglobin level and oxygen saturation. LED_1_ = LED (light-emitting diode) at 750 nm (*λ*_1_); LED_2_ = LED at 830 nm (*λ*_2_); distance between the light-dispatching point (light-emitting diode; LED) and the light-receiving point (photo diode; PD) = 5 mm for fingertip sensor and 30 mm for the anterior abdominal region and lumbar region sensor; *l*_0_ *λ* = intensity of the incident light with a wavelength of *λ* at time *t*; *lλ*(*t*) = intensity of the scattered light with a wavelength of *λ* at time *t*.

A tissue hemoglobin monitor (PAS-500) was used for the measurement. The sensors shown in Figure 3 were used for measuring the anterior abdominal muscle tissue total hemoglobin level, the anterior abdominal muscle tissue oxygen saturation, the lumbar muscle tissue total hemoglobin level, the lumbar muscle tissue oxygen saturation, the fingertip skin tissue total hemoglobin level, and the fingertip skin tissue oxygen saturation. 

Measurement was based on the following principle. First, the absorbance of reflected light in the target region was measured both during compression (time *t*_0_) and after the end of compression (*t*_1_). From the absorbances at each of these two time points, the pulsatile fraction (the fraction reflecting arterial blood fluctuation) was subtracted with a high-range filter, to yield *A*_*λ*_(*t*_0_) and *A*_*λ*_(*t*_1_). Modified Lambert-Beer rule was then applied to the difference in absorbance between *t*_1_ and *t*_0_, that is, (*A*_*λ*_(*t*_1_)−  *A*_*λ*_(*t*_0_)), to determine the tissue venous blood oxygen saturation [11–13]. A double-adhesive tape was used to fix the sensor (applied to the palmar side of the index finger of the nondominant arm), the upper part of the left-sided pad of the HSG sheet belt (applied to the anterior abdominal region, i.e., the center of the anterior abdominal region around the umbilicus), and the upper part of the left-sided pad of the HSG sheet belt (applied to the lumbar region, i.e., the area bounded by the lowest ribs of either side above and the iliac crests of either side below). The physiological zero level of the tissue hemoglobin monitor was determined on the basis of the stabilized baseline value after compression of the test site with the sensor to drive blood out from the tissue. The difference between the measured tissue total hemoglobin level and the physiological zero was adopted as the tissue total hemoglobin level [14]. The results of the measurements were represented graphically as the percent value relative to the value at rest (recorded 20 minutes after the start of the test).

### 2.6. R-R Interval Variability Spectrum on the Electrocardiogram

Electrocardiographic (ECG) recording was carried out with the V5 chest lead (Polymate AP1524, Digitex Lab. Co., Ltd., Tokyo, Japan). Trigger pulses synchronized with the R wave peak were fed into a computer for calculation of the R-R interval to a precision of 1 msec. Spectral analysis of heart rate variability was carried out by subjecting the R-R interval data to power spectral analysis using the maximum entropy method, and the power spectral density (PSD: ms^2^/Hz) in the 0.04–0.40 Hz range was determined. The total power spectral density in the 0.04–0.15 Hz range was deemed as the LF (low-frequency component; ms^2^), and that in the 0.15–0.40 Hz range was deemed as the HF (high-frequency component; ms^2^). In accordance with the methods reported by Akselrod et al., Pagani et al., Berger et al., and Saul et al., the LF/HF ratio of the R-R interval change was determined as an indicator of sympathetic nerve activity, and the HF was determined as an indicator of parasympathetic nerve (vagus) activity [15–18]. LF and HF were then normalized by dividing them by the total power (LF + HF) [19] and designated as LF norm and HF norm, respectively. Both LF/HF and HF norm were averaged over a 5-minute period.

### 2.7. Electrogastrography

**Figure 4:**
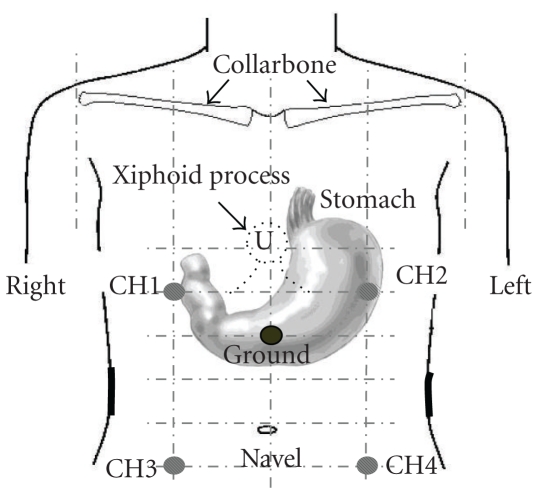
Sites of electrode placement for the electrogastrography. An indifferent electrode was set at the midpoint between the sternal xiphoid process and the umbilicus along the median line. Electrode 1 (CH1) was set at the midpoint between the sternal xiphoid process and the indifferent electrode along the right midclavicular line. Electrode 2 (CH2) was set at the midpoint between the sternal xiphoid process and the indifferent electrode along the left midclavicular line. Electrode 3 (CH3) was set at the lower 1/2 of the segment between the indifferent electrode and the umbilicus along the right midclavicular line. Electrode 4 (CH4) was set at the lower 1/2 of the segment between the indifferent electrode and the umbilicus along the left midclavicular line.

From the day before electrogastrography, the subjects were strictly prohibited from taking any drug, drinking alcohol, and smoking, and at the supper, fibrous food was avoided. After 9:00 p.m. of the day before the test, no food was taken, and only water was permitted as a beverage. In addition, to avoid the influence of residual food in the stomach on the day of the test, two bars of Calorie Mate (200 kcal, Otsuka Pharmaceut. Co., Ltd. Tokyo, Japan) as low-residue food and vegetable juice (Yasai Seikatu 100 Original: Kagome Co., Ltd., Aichi, Japan) were taken by 2 hours and 30 minutes before the initiation of the test. The subjects arrived at the research institution before 8:00 a.m., measurement was initiated at about 9:00 a.m., and eating and drinking were prohibited until the completion of the test (about two hours later). Electrogastrography was performed with the subject sitting at an angle of 60 degrees. Concerning the positioning of electrodes for electrogastrography, recording from the site that is the closest to the stomach was considered to be appropriate according to the report by Okuno et al. [20]. However, there are marked individual differences in the location and morphology of the stomach. Therefore, to obtain electrogastrograms straightforwardly, spectrum analysis was performed under specified conditions for frequency and amplitude extraction. As a result, 1 and 2 ch recordings were excellent. In addition, as a result of the isopower mapping of electrogastrograms after total gastrectomy or total colectomy performed by Homma et al., 3 and 4 ch recordings were considered to be appropriate for colon recording [21]. As shown in Figure 4, an indifferent electrode was placed at the midpoint on the median line connecting the xiphoid process and the umbilicus, and the recording electrodes (Disposable Electrodes Viltrode P, Nihon Kohden Corporation, Tokyo, Japan) were placed in the upper 1/4th segment and lower 1/4th segment of the right and left midclavicular lines between the sternal xiphoid process and the umbilicus, and a 4-channel electrogastrography was carried out.

### 2.8. Analysis of the Electrogastrograms

Percutaneous electrogastrography (EGG) was carried out using an electrogastrographic recorder (EG, Nipro Corporation, Osaka, Japan). To avoid contamination by respiration-related signals, an 8th order high-range filter (9.0 cpm: cycle/minute) was used. To eliminate drift murmurs associated with body motion and baseline changes, the signals were passed through a third-order low-range filter (1.5 cpm). The EGG signals were thus amplified and recorded at a sampling rate of 1 Hz. MBFA (multiple bandpass filter analysis) [22] was employed for the analysis. Seventy-five 0.1-cpm wide bandpass filters were set for the 1.5–9.0 cpm range. Spectral analysis of the serial changes in the amplitude was carried out in the 3 cpm (2.8–3.2 cpm) range obtained from the potential gradient between 1 ch and 2 ch and on the 6 cpm (5.8–6.2 cpm) range from the potential gradient between 3 ch and 4 ch. Of these ranges, the 3 cpm range represents the gastric electrical activity, called the “slow wave,” which appears with spontaneous regularity during hunger (the pacemaker is located along the greater curvature of the upper 1/3 of the gastric body, and this activity governed by the vagus nerve is produced by the interstitial cells of Cajal). The 6 cpm range represents the electrical activity of the colon. MBFA is useful for the spectral analysis of data from short-term measurements, since it allows a spectrum to be obtained from the entire range measured, even with small amounts of data. With the PSD (power spectral density) of the amplitude at rest (recorded 5–10 minutes before application of the HSG sheet) serving as the criterion level, analysis was conducted of the changes from the 15th to 20th minute, 35th to 40th minute, and 50th to 55th minute during and after application of the sheet, and from the 23rd to 28th minute after removal of the sheet (each sampling size: 300 points).

### 2.9. Statistical Analysis

The values of all parameters were expressed as mean ± SE. Significances of differences were tested by Tukey-Kramer's multiple comparisons. *P* < .05 was regarded as denoting statistical significance.

## 3. Results

### 3.1. Changes in the Feeling of Comfort/Discomfort in the Abdomen and Perception of Thermal Sensation

**Figure 5:**
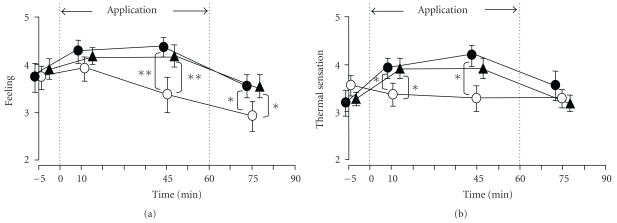
Time course of changes in the feeling of comfort/discomfort in the abdomen and in the thermal sensation at the fingertips during and after application of the HSG sheet. ∘; Control (only the waist belt applied to the anterior abdominal region or lumbar region), ●; HSG sheet applied with a waist belt to the anterior abdominal region, ▴; HSG sheet applied with a waist belt to the lumbar region. (a) Feeling of comfort/discomfort in the abdomen. (b) thermal sensation at the fingertips. Both the feeling of comfort/discomfort in the abdomen and the thermal sensation at the fingertips were scored on a five-category scale: (a) (comfortable: 5, slightly comfortable: 4, neutral: 3, slightly uncomfortable: 2, and uncomfortable: 1), (b) (warm: 5, slightly warm: 4, neutral: 3, slightly cold: 2, and cold: 1). Both were scored after 20 minutes of acclimation, at 10 and 45 minutes during and after application of the HSG sheet belt or sham belt (the period marked with dotted line in Figure 5), and at 15 minutes after removal of the belt. Data show the mean ± SE of scores from 10 subjects. *(*P* < .05) and **(*P* < .01) indicate significant differences from the control group (Tukey-Kramer's multiple comparison).

As shown in Figure 5(a), the mean score for the feeling of comfort/discomfort in the abdomen following application of the sham belt (waist belt) to the anterior abdominal region or lumbar region (the control group) decreased gradually over time, while after the application of the HSG sheet belt, the mean score rose slightly at 45 minutes after the application and 15 minutes after the removal (HSG-sheet group). These differences from the control group were statistically significant (*P* < .01 and *P* < .05). As shown in Figure 5(b), the score for the fingertip thermal sensation rose after application of the HSG sheet. As compared to the control group, the score rose significantly at 10 and 45 minutes during and after application of the HSG sheet to the anterior abdominal region or the lumbar region (*P* < .05). These results indicate that application of the HSG sheet to the anterior abdominal region or the lumbar region increases the feeling of comfort in the abdomen and improves the thermal sensation at the fingertips.

### 3.2. Measurement of the Tissue Temperature

After the subjects sat reclined at an angle of 60 degrees for 20 minutes to become acclimatized to the experimental room conditions controlled at a temperature of 23.0°C and relative humidity of 45.0%, the mean skin temperature in various parts of their body were determined to be as follows: anterior abdominal region, 34.65 ± 0.31°C; lumbar region, 34.32 ± 0.21°C; fingertips, 33.73 ± 0.29°C; instep, 33.19 ± 0.71°C. The mean subcutaneous tissue temperature in the anterior abdominal region and lumbar region was 35.83 ± 0.20°C and 36.17 ± 0.19°C, respectively.

**Figure 6:**
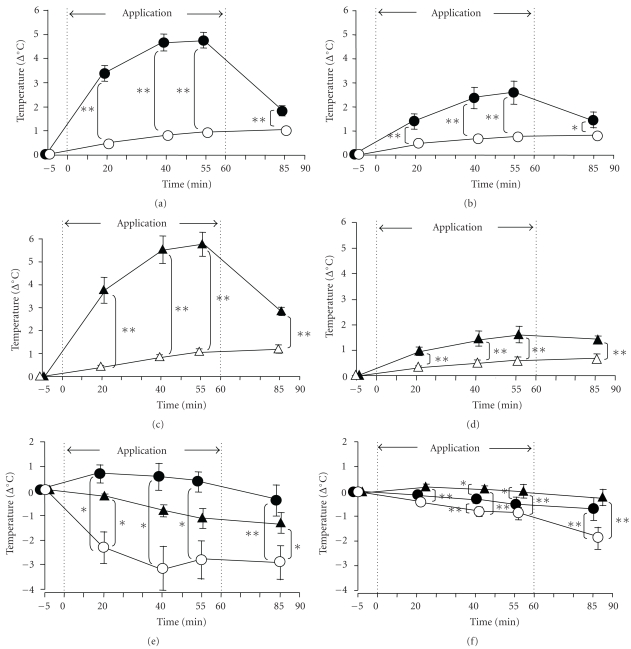
Serial changes of the tissue temperature during and after application of the HSG sheet. ° (anterior abdominal region) or ∆ (lumbar region); control group (sham belt), ●; HSG sheet applied and fixed as a waist belt to the anterior abdominal region, ▴; HSG sheet applied and fixed as a waist belt to the lumbar region, abdominal region (a); skin temperature beneath the HSG sheet, abdominal region (b); subcutaneous tissue temperature, lumbar region (c); skin temperature beneath the HSG sheet, lumbar region (d); subcutaneous tissue temperature, periphery (e); fingertip skin temperature, periphery (f); instep skin temperature. The difference between the tissue temperature at rest (=0°C) and the tissue temperature during the 60-minute period of application of the sham belt or HSG sheet belt to the anterior abdominal region or lumbar region (the period marked with dotted line) and the 30-minute period after removal of the sham belt or HSG sheet belt (until 90 minutes during and after application of the belt/sheet) are shown. Data are mean ± SE of the 10 subjects. *(*P* < .05) and **(*P* < .01) indicate significant differences from the control group (Tukey-Kramer's multiple comparison).

The skin temperature in the anterior abdominal region immediately beneath the HSG sheet was significantly increased at 20, 40, and 55 minutes during and after application of the HSG sheet (which produces heat and steam at 38.5 °C) to the anterior abdominal region (*P* < .01) and remained high at 25 minutes after the removal of the sheet (*P* < .01) (Figure 6(a) Abdominal region). 

Significant increase of the subcutaneous tissue temperature was noted in the vicinity of the HSG sheet application area on the anterior abdominal region at 20, 40, and 55 minutes after the application of the HSG sheet, and also at 25 minutes after its removal, as compared with the corresponding values in the control group in which a sham belt was applied to the anterior abdominal region/lumbar region (*P* < .05, *P* < .01) (Figure 6(b) Abdominal region). 

Significant increase of the skin temperature immediately beneath the HSG sheet was observed at 20, 40, and 55 minutes during and after application of the HSG sheet to the lumbar region (*P* < .01), as also at 25 minutes after the sheet removal (*P* < .01) (Figure 6(c) Lumbar region).

A tendency towards increase of the subcutaneous tissue temperature in the lumbar region was observed during and after application of the HSG sheet to the lumbar region, and the values at 20, 40, and 55 minutes after the application of the sheet and also at 25 minutes after its removal were significantly higher than the corresponding values in the control group (*P* < .01) (Figure 6(d) Lumbar region). These results clearly indicate that, as expected, application of the HSG sheet to the anterior abdominal region or lumbar region elevated the skin temperature immediately beneath the HSG sheet. In addition, the temperature of the deeper tissues (up to a depth of about 10 mm in the underlying subcutaneous tissue) in the vicinity of the sheet-applied area was also increased (Figure 6(b) abdominal region and Figure 6(d) lumbar region). 

Changes of the temperature in the peripheral tissues, such as at the fingertips and instep, as representatives of local tissues, following application of the HSG sheet to the anterior abdominal region or lumbar region are shown in Figures 6(e) and 6(f) periphery and 

The temperature of these tissues in the control group decreased gradually with time because of the ambient room temperature of 23.0°C. Significant increase of the differences in the temperature at the fingertips (Figure 6(e) Periphery), and to a lesser extent that at the instep (Figure 6(f) Periphery), was noted between the control and HSG sheet group at 20, 40, and 55 minutes during and after application of the HSG sheet to the lumbar region (*P* < .01) and also at 25 minutes after its removal (*P* < .01). The differences in the temperature at the fingertips between the control and HSG-sheet groups were more pronounced in the subjects with the sheet applied to the anterior abdominal region than in those with the sheet applied to the lumber region. Significant differences in the fingertip and instep skin temperature between the two groups were still observed at 25 minutes after the removal of the HSG sheet (*P* < .05).

These results suggest that the HSG sheet applied to the anterior abdominal region or lumbar region prevented reduction of the fingertip skin temperature. A tendency towards prevention of reduction of the instep skin temperature was also noted, at 40 minutes after the sheet application (*P* < .01) and at 25 minutes after its removal (*P* < .01) (Figure 6(f) periphery). These findings of the skin temperature well reflected the effect of the sheet on the scores for thermal awareness in the subjects, shown in Figure 1.

### 3.3. Serial Changes in the Tissue Total Hemoglobin Levels and Oxygen Saturation

**Figure 7:**
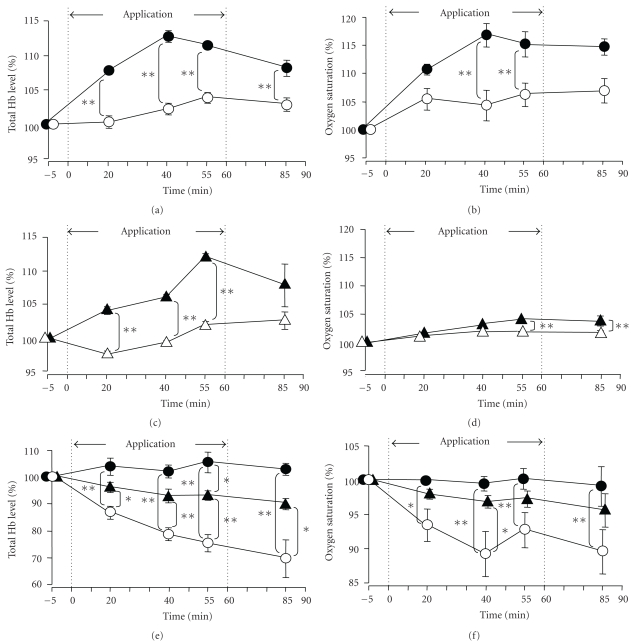
Serial changes of the tissue total hemoglobin levels and oxygen saturation during and after application of the HSG sheet. ∘ (anterior abdominal region) or ∆ (lumbar region); control group (sham belt), ●; HSG sheet applied and fixed as a waist belt to the anterior abdominal region, ▴; HSG sheet applied and fixed as a waist belt to the lumbar region, abdominal region (a); abdominal muscle tissue total hemoglobin level, abdominal region (b); abdominal muscle tissue oxygen saturation, lumbar region (c); lumbar muscle tissue total hemoglobin level, lumbar region (d); lumbar muscle tissue oxygen saturation, periphery (e); total hemoglobin level in the fingertip skin tissue, periphery (f); fingertip skin tissue oxygen saturation. Relative to the tissue total hemoglobin levels or tissue oxygen saturation at rest (=100%), the percentages of the tissue total hemoglobin level and tissue oxygen saturation during the 60-minute period of application of the sham belt or HSG sheet belt to the anterior abdominal region/lumbar region (the period marked with dotted line) and the 30-minute period after the removal of the sham belt or HSG sheet belt (until 90 minutes during and after application of the belt/sheet) are shown. Data are mean ± SE of the 10 subjects. *(*P* < .05) and **(*P* < .01) indicate significant differences from the control group (Tukey-Kramer's multiple comparison).

Significant increase of the muscle tissue total hemoglobin levels in the area of application of the sheet was noted at 20, 40, and 55 minutes during and after application of the HSG sheet to the anterior abdominal region (*P* < .01), as also at 25 minutes after its removal (*P* < .01), as compared with the corresponding values in the control group in which the sham belt was applied (Figure 7(a) abdominal region). A tendency towards increase of the muscle tissue blood oxygen saturation in the anterior abdominal region at the site of the sheet application was noted at various measurement points during and after application of the HSG sheet to the anterior abdominal region, and the increases noted at 40 and 55 minutes after the sheet application were significant (*P* < .01) (Figure 7(b) abdominal region). At 25 minutes after the sheet removal; however, no significant difference of the oxygen saturation was observed between the HSG sheet and control groups. Significant increase of the muscle tissue total hemoglobin levels in the lumbar region was also noted at 20, 40, and 55 minutes during and after application of the HSG sheet to the lumbar region (*P* < .01) (Figure 7(c) lumbar region), while the significant difference was no longer observed at 25 minutes after the sheet removal. A tendency towards rise of the muscle tissue oxygen saturation in the lumbar region was noted during and after application of the HSG sheet to the lumbar region, and the increases at 55 minutes after the sheet application (*P* < .01) and at 25 minutes after the sheet removal (*P* < .01) were significant. These results show that application of the HSG sheet to the anterior abdominal region or lumbar region also elevated the muscle tissue total hemoglobin levels and muscle tissue oxygen saturation in the vicinity of the HSG sheet application area, consistent with the increase of the tissue temperatures illustrated above (Figure 6 abdominal region and lumbar region).

Next, we performed analysis of the serial changes of the skin tissue total hemoglobin levels and oxygen saturation in the periphery, using the fingertips as a representative region. The values of these parameter in the control group with the sham sheet decreased gradually with time, probably because of the ambient temperature in the room of 23.0°C (Figures 7(e) and 7(f) Periphery). Application of the HSG sheet to the anterior abdominal region or the lumbar region prevented the reduction of these parameters at the fingertips. Therefore, significant differences of these parameters between the control and HSG sheet application groups were observed at 20, 40, and 55 minutes during and after application of the HSG sheet to the anterior abdominal region (*P* < .01), as well as at 25 minutes after its removal (*P* < .01) (Figure 7(e) Periphery). Application of the HSG sheet to the lumbar region also prevented reduction of the fingertip skin tissue hemoglobin levels, with significant increases in the difference of the fingertip skin tissue total hemoglobin level between the control and HSB sheet groups observed at 20, 40, and 55 minutes after the sheet application (*P* < .05, *P* < .01), and also at 25 minutes after its removal (*P* < .05). The significant difference of the finger total hemoglobin levels between the control and HSG sheet groups at 55 minutes was more pronounced in the subjects with the sheet applied to the anterior abdominal region than in those with the sheet applied to the lumbar region (*P* < .05). Furthermore, a tendency towards higher differences was noted at all the measurement time-points until 85 minutes.

 Following the application of the HSG sheet to the lumbar region, the difference in the fingertip oxygen saturation values between the control and HSG sheet groups showed a tendency towards increase with time, and the increase in difference at 40 minutes after the sheet application was significant (*P* < .05). At 25 minutes after the sheet removal, however, the difference was no longer statistically significant. These results suggest that application of the HSG sheet to the anterior abdominal region or the lumbar region prevented the reduction over time of the fingertip oxygen saturation value. In addition, the prevention of decrease of the fingertip skin tissue total hemoglobin levels by application of the HSG sheet was more pronounced when the sheet was applied to the anterior abdominal region than when it was applied to the lumbar region.

### 3.4. Serial Changes in the R-R Interval Variability Spectrum on the Electrocardiogram

**Figure 8:**
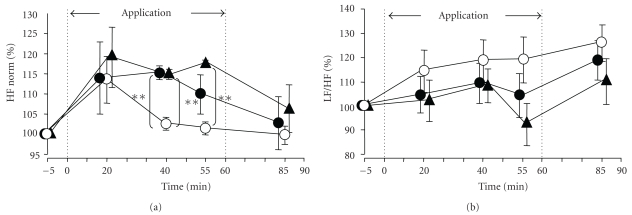
Serial changes in the electrocardiographic R-R interval variability spectrum during and after application of the HSG sheet. ∘; Control (sham belt applied to the anterior abdominal region and lumbar region), ●; HSG sheet applied and fixed as a waist belt to the anterior abdominal region, ▴; HSG sheet applied and fixed as a waist belt to the lumbar region, (a) HF norm (high-frequency component: an index of parasympathetic activity), (b) LF/HF (ratio of the low and high-frequency components of the power spectrum of the HR variability: an index of sympathetic activity). Relative to the level of each parameter at rest (=100%), the percentages of each during the 60-minute period of application of the sham belt or HSG sheet belt to the anterior abdominal region/lumbar region (the period marked with dotted line) and the 30-minute period after removal of the sham belt or HSG sheet belt (until 90 minutes during and after application of the belt/sheet) are shown. Data are mean ± SE of the 10 subjects. **(*P* < .01) indicates significant differences from the control group (Tukey-Kramer's multiple comparison).

A tendency towards increase of the HF norm (an indicator of parasympathetic activity) was noted following application of the HSG sheet to the anterior abdominal region, and the increase at 40 minutes after the sheet application was significant (*P* < .01) (Figure 8(a)), as compared with the value at the corresponding time point in the control group in which the sham belt was applied to the anterior abdominal region or the lumbar region. At 25 minutes after the sheet removal, there was no significant difference in the value of this parameter as compared with that in the control group. When the HSG sheet was applied to the lumbar region, a tendency towards increase of the HF norm after the sheet application was noted, and the increase at 40 and 55 minutes after the sheet application was significant (*P* < .01). At 25 minutes after the sheet removal from the lumbar region, there was no significant difference in the value of this parameter as compared with that in the control group. These results suggest that application of the HSG sheet to the anterior abdominal region or the lumbar region elevates the HF component of HRV. A tendency towards decrease of the LF/HF ratio (an indicator of sympathetic activity) was noted (Figure 8(b)), following HSG sheet application although this change was not significant as compared with that in the control group.

### 3.5. Serial Changes in the Electrogastrographic Parameters

**Figure 9:**
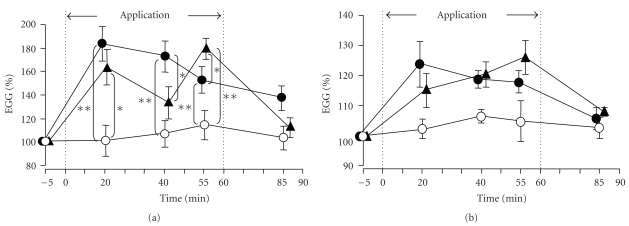
Serial changes in the electrogastrographic parameters during and after application of the HSG sheet. ∘; Control (sham belt applied to the anterior abdominal region and lumbar region), ●; HSG sheet applied and fixed as a waist belt to the anterior abdominal region, ▴; HSG sheet applied and fixed as a waist belt to the lumbar region, (a) parameter at 3 cpm (2.8–3.2 cycle/min: an indicator of the gastric electrical activity), (b) parameter at 6 cpm (5.8–6.2 cycle/min: an indicator of the colonic electrical activity). Relative to the level of each parameter at rest (=100%), the percentages of each during the 60-minute period of application of the sham belt or HSG sheet belt to the anterior abdominal region and/or lumbar region (the period marked with dotted line) and the 30-minute period after removal of the sham belt or HSG sheet belt (until 90 minutes during and after application of the belt/sheet) are shown. Data are mean ± SE of the 10 subjects. *(*P* < .05) and **(*P* < .01) indicate significant differences from the control group (Tukey-Kramer's multiple comparison).

Significant increase of the PSD (power spectral density) for the 3 cpm range, an indicator of gastric electrical activity, was noted (Figure 9(a)), at 20, 40, and 55 minutes after HSG sheet application to the anterior abdominal region (*P* < .01) as compared with the corresponding values in the control group, in which the sham belt was applied to the anterior abdominal region or the lumbar region. However, there was no significant difference in the value of this parameter as compared with that in the control group at 25 minutes after the sheet removal from the anterior abdominal region. Thus, the response to the HSG sheet application disappeared rapidly. When the HSG sheet was applied to the lumbar region, significant increase of the PSG for 3 cpm was noted at 20 and 55 minutes after the sheet application (*P* < .05, *P* < .01). At 25 minutes after the removal of the sheet from the lumbar region, however, there was no significant difference in the value of this parameter as compared with that in the control group, similar to the finding for the sheet application to the anterior abdominal region. The PSD for 3 cpm at 40 minutes was significantly lower following application of the HSG sheet to the lumbar region than after that following its application to the anterior abdominal region (*P* < .05). This parameter at 55 minutes was significantly higher after the sheet application to the lumbar region than that following its application to the anterior abdominal region (*P* < .05). At 25 minutes after the sheet removal, however, there was no significant difference in the value of this parameter between the HSG sheet and control groups. These results suggest that application of the HSG sheet to the anterior abdominal region or the lumbar region elevates the PSD for 3 cpm. A tendency towards elevation of the PSD for the 6 cpm range, an indicator of colonic electrical activity, was also noted (as shown in Figure 9(b)) following HSG sheet application to the anterior abdominal region or lumbar region; however, this change was not significant.

## 4. Discussion

As some reports, such as that by Hosono et al., have reported favorable data on the effects of warming devices in women suffering chronic constipation [2], we examined the effects of a recently developed HSG sheet by analyzing subjects' physiological parameters.

In preliminary tests during and after the application of the HSG sheet to healthy volunteers for 60 min, the skin temperature underneath the sheet was elevated by 1°C -2°C, as expected (data not shown). Moreover, the application of the sheet prevented the peripheral fingertip skin temperature decreasing (because the room temperature was 23.0°C), and blood flow increased. Therefore, we decided to examine more precisely the effects of the HSG sheet in this study.

As shown in Figure 5, the scores for a feeling of comfort/discomfort in the abdomen following the application of the HSG sheet in women with a tendency towards constipation was improved to feeling comfortable. This result suggests that the application of the sheet could have a favorable effect on patients who suffer from discomfort in the abdomen region, such as those who have undergone abdominal surgery. We, therefore, intended to objectively examine its effects.

In the present experiment, in which the HSG sheet was applied for 60 min, the temperature of the skin just beneath the HSG sheet application area on the anterior abdominal region or lumbar region increased over time to almost 38.0°C; therefore, the sheet was concluded to be a useful device for warming. Furthermore, application of the sheet increased the peripheral skin temperature, which would be expected to result in an increase in blood flow in the peripheral tissues. Therefore, we consider that application of the HSG sheet improves peripheral circulatory hemodynamics. Considering that skin temperature is a useful indicator of skin blood flow [23], the results of this study suggest that in addition to producing an increase in the local skin blood flow in the application area, HSG sheet application to the anterior abdominal region or lumbar region also increases the peripheral skin blood flow even in remote parts of the body, such as the fingertips and instep.

While skin blood flow may continue to increase to a certain degree, the skin temperature reaches a plateau earlier, making estimates of skin blood flow calculated from skin temperature unreliable [23]. We, therefore, conducted further analysis of the tissue total hemoglobin level as an indicator of tissue blood flow, because tissue total hemoglobin levels have been shown to be positively correlated with tissue blood flow in the absence of congestion [24–26]. The significant increases in the muscle tissue total hemoglobin level and the muscle tissue oxygen saturation in the vicinity of the sheet application area noted in this study suggest that application of the sheet also increases muscle tissue blood flow in the vicinity of the sheet application area. On the other hand, application of the HSG sheet prevented a decrease in local skin total hemoglobin levels and oxygen saturation at the periphery (fingertips), suggesting that application of the sheet also prevented a decrease in the blood flow to the fingertips. In addition, we found that the prevention of decreases in skin tissue total hemoglobin levels at the fingertips achieved by the application of the HSG sheet, which reflects its effects on local blood flow, was more pronounced when the sheet was applied to the anterior abdominal region than when it was applied to the lumbar region. The reason for the differences in the effect is not known at the moment.

With regard to the results of the spectral analysis of electrocardiogram R-R interval variability, application of the HSG sheet resulted in a significant increase in the HF norm and a tendency towards a decrease in the LF/HF ratio. The HF component corresponds to respiratory sinus arrhythmia and is considered to serve as a quantitative indicator of cardiac vagal activity (tonus) [18]. The LF/HF ratio is inversely proportional to the HF. Since the LF component is mediated by both cardiac vagal activity and sympathetic activity, at least part of the increase in the LF/HF ratio probably reflects a reduction in cardiac vagal activity and an increase in sympathetic activity [27]. However, the results of such analyses are known to be affected by respiration [17]. For example, an increase in the respiratory rate and a decrease in the tidal volume would reduce the HF component. While the LF component is not affected by the respiratory rate, the LF/HF ratio would be reduced by prolongation of the respiratory cycle. As the present study was carried out under spontaneous respiration without strict regulation of respiratory activity (control group: 17.36 ± 0.90 count/min, HSG-Ab: 17.41 ± 0.98 count/min, HSG-Lum: 17.33 ± 0.94 count/min), it was not possible to completely eliminate the influence of respiration on these parameters. However, considering that no major changes in respiratory activity (e.g., a reduction of the respiratory rate to 0.15 Hz or less or a reduction of the cardiac beat/respiration ratio to 2 or less) were noted during the study, it would appear that the application of the HSG sheet causes predominance of the parasympathetic activity.

In our analysis of the electrogastrograms, the application of the HSG sheet resulted in a significant increase in the PSD in the 3 cpm range; however, no significant change in the PSD was observed in the 6 cpm range although it tended to increase following the application of the HSG sheet. Percutaneous recording of the periodic electrical activity of the stomach via the abdominal wall was first attempted in 1922 by Alvarez [28]. Since then, this technique has been used for electrophysiological analyses of gastrointestinal motility (percutaneous electrogastrography). The pacemaker for this activity is located along the greater curvature of the upper 1/3 of the gastric body, and this spontaneous periodic electrical activity is governed by the vagus nerve originating from the interstitial cells of Cajal [29]. Homma et al. reported that the PSD in the 3 cpm range recorded from the upper abdominal region and the PSD in the 6 cpm range recorded from the lower abdominal region reflect gastric and colonic electrical activity, respectively [21]. Pezzolla et al. reported that the spectral power peaks at frequencies of about 3, 6, and 10 cpm represent frequency components associated with the activity of the stomach, large bowel, and small bowel, respectively [30]. Imai et al. reported that gastric electrical activity is predominantly regulated by the autonomic nervous system under parasympathetic predominance for the following reasons: atropine sulfate (a muscarinic receptor blocker) suppressed gastric motility and gastric acid secretion and reduced EGG amplitude, whereas neostigmine (a cholinesterase inhibitor) stimulated gastric function and increased EGG amplitude [31]. When these previous findings are considered along with the findings of the present study in which application of the HSG sheet significantly increased the PSD in the 3 cpm range, it seems likely that the application of the HSG sheet stimulates gastric motility and causes predominance of the parasympathetic activity. The tendency towards an increase of the PSD in the 6 cpm range observed after HSG sheet application suggests that the application of this sheet may also increase large bowel motility. Further studies are required to clarify these findings.

Following application of the HSG sheet to the anterior abdominal region or the lumbar region, the temperature stimulus is probably converted by the peripheral sensory nerves into electrical signals (action potentials) and then transmitted to the CNS. As the molecules involved in temperature perception, we may consider some transient receptor potential (TRP) channel, such as TRPV3 and TRPV4. This view is based on the report that these channels were activated in the high temperature range of over 30°C (TRPV3 > 32–39°C; TRPV4 > 27–35°C) [32]. Furthermore, since these channels are also expressed by keratinocytes on the skin surface [33, 34], it is possible that the production of heat (about 38.5°C) at a lower level than that produced by conventional hot packs in the anterior abdominal region or lumbar region could induce these channels to exert physiological effects in the present study.

Taken together, these results suggest that the application of the HSG to the anterior abdominal region or lumbar region improves the peripheral hemodynamics and gastric motility, suppresses sympathetic activity, and promotes parasympathetic predominance. 

Although the application of the HSG sheet in the present study was only performed for one short session (1 hour), it resulted in an increase in peripheral blood flow, parasympathetic activity predominance, improvement in the subjects' sense of well-being, and a warm sensation in their periphery. These effects, which were also seen during chronological analysis, suggest that the HSG sheet is useful for alleviating chronic constipation and improving gastrointestinal function. Following this finding, we plan to develop protocols for the long-term application of the HSG sheet (application of the sheet for a certain number of hours per day for a certain period, etc.) with the ultimate goal of developing a device capable of alleviating chronic constipation and gastrointestinal dysfunction.

## 5. Conclusion

We concluded that the application of the HSG sheet improves peripheral hemodynamics and autonomic regulation, induces a feeling of comfort in the abdomen, and provides a beneficial environment for the improvement of gastrointestinal movements. The effects would improve the quality of life of patients who have undergone abdominal surgery or who are suffering from constipation.
